# Comparing GIS-Based Measures in Access to Mammography and Their Validity in Predicting Neighborhood Risk of Late-Stage Breast Cancer

**DOI:** 10.1371/journal.pone.0043000

**Published:** 2012-08-28

**Authors:** Min Lian, James Struthers, Mario Schootman

**Affiliations:** Department of Medicine, Washington University School of Medicine, St. Louis, Missouri, United States of America; University of Campinas, Brazil

## Abstract

**Background:**

Assessing neighborhood environment in access to mammography remains a challenge when investigating its contextual effect on breast cancer-related outcomes. Studies using different Geographic Information Systems (GIS)-based measures reported inconsistent findings.

**Methods:**

We compared GIS-based measures (travel time, service density, and a two-Step Floating Catchment Area method [2SFCA]) of access to FDA-accredited mammography facilities in terms of their Spearman correlation, agreement (Kappa) and spatial patterns. As an indicator of predictive validity, we examined their association with the odds of late-stage breast cancer using cancer registry data.

**Results:**

The accessibility measures indicated considerable variation in correlation, Kappa and spatial pattern. Measures using shortest travel time (or average) and service density showed low correlations, no agreement, and different spatial patterns. Both types of measures showed low correlations and little agreement with the 2SFCA measures. Of all measures, only the two measures using 6-timezone-weighted 2SFCA method were associated with increased odds of late-stage breast cancer (quick-distance-decay: odds ratio [OR] = 1.15, 95% confidence interval [CI] = 1.01–1.32; slow-distance-decay: OR = 1.19, 95% CI = 1.03–1.37) after controlling for demographics and neighborhood socioeconomic deprivation.

**Conclusions:**

Various GIS-based measures of access to mammography facilities exist and are not identical in principle and their association with late-stage breast cancer risk. Only the two measures using the 2SFCA method with 6-timezone weighting were associated with increased odds of late-stage breast cancer. These measures incorporate both travel barriers and service competition. Studies may observe different results depending on the measure of accessibility used.

## Introduction

Breast cancer is an important public health issue and accounts for about 28% of cancer incidence and 15% cancer mortality in the United States [1]. Screening mammography reduces the risk of breast cancer death by early detection [2]. Geographic barriers in access to healthcare could significantly impact on population health. In recent years, it has become common to investigate the influence of geographic distribution of mammography service on mammography screening use and stage at diagnosis of breast cancer. However, findings from previous reports vary to a great degree. Some studies, found that barriers in spatial accessibility to mammography facilities increased the risk of non-adherence to screening and/or stage at diagnosis of breast cancer [3], [4], [5], [6], [7], [8], but other studies did not [9], [10], [11]. Regardless of the limitations and potential biases in study design and data collection, inconsistency in these findings might result from the use of varying spatial methods in assessing access to mammography. Few studies have compared differences in Geographic Information System (GIS)-based measures of accessibility.

Previous assessments of spatial accessibility to mammographic service include neighborhood availability (or service density – the number of facility per population) [3], [4], [5], [9] and travel distance (or travel time) to the nearest facility [10], [11], [12], [13], [14]. Service density has been frequently used and is easy to compute. The use of a road-network-based travel distance/time is becoming a popular measure with the rapid development of available GIS techniques. However, both of these two measures have limitations. The former ignored the interaction between population and service facilities across arbitrary neighborhood boundaries, while the latter does not account for the competition among different service facilities (demand) [15]. A gravity model overcomes both limitations through integration of travel barriers and service competitions and has become an alternative approximation of spatial accessibility to mammography services. Gravity models have been used extensively by geographers, but have been underutilized by epidemiologists. Two important gravity models are the Kernel Density (KDE) [16] method and the two-step floating catchment area (2SFCA) method [17]. The major limitation of the KDE method is that it ignores travel barriers by using a straight line Euclidean distance. The 2SFCA method uses the actual road network distance, which is much closer to real-world situations. Recently, a zonal- or continuous- weighting parameter was added to this method which allowed for a distance-related decay. This resulted in an enhanced two-step floating catchment area (E2SFCA) method [18] and a Gaussian two-step floating catchment area (G2SFCA) method [19]. More recently, the 2SFCA method was further improved to overcome the influences of rural-urban difference or large irregular study area through using varied catchment sizes [20] or aggregating small-area 2SFCA measures to larger neighborhoods [21]. Due to technical difficulties in implementing, the 2SFCA method is still underutilized in epidemiology.

In this study, we compared these three methods (nine GIS-based measures) in assessing access to mammography facilities at the block group-level in the St. Louis area. In a previous study, we found the risk of advanced breast cancer was higher in the St. Louis area than elsewhere in Missouri [22]. As an indicator of predictive validity, we also compared the associations of these nine measures with neighborhood risk of late-stage breast cancer after adjusting for demographics and neighborhood socioeconomic deprivation using cancer registry data.

## Materials and Methods

### Study Population

The study area includes St. Louis City and St. Louis County, Missouri, that is located in the center of the greater St. Louis Metropolitan area, covering 590 square miles including 1124 block groups according to the 2000 Census. There are 719,737 women living in both counties, 337,966 of which are age 40 and above. Note that St. Louis City is its own county in Missouri. We obtained 2002–2006 primary breast cancer incidence cases from the Missouri Cancer Registry. Using a GIS, the address of breast cancer cases was geocoded to corresponding Census block groups and matched to U.S. Census 2000 TIGER/Line files. Breast cancer stage was defined according to the AJCC staging system as ductal/lobular carcinoma *in situ* (DCIS/LCIS, stage 0) and invasive breast cancer (stages I, II, III and IV). The study outcome was dichotomized as late-stage breast cancer (stages II–IV) vs. early-stage breast cancer (stages 0-I). Age was categorized as younger than 50 years, 50–64 years, and age 65 and above. Race was grouped as non-Hispanic White, African American, and Other. After excluding 62 ungeocoded cases and 148 cases with missing stage, a total of 4205 breast cancer cases were included in the analysis. This study was approved by Washington University's Institutional Review Board.

### GIS-Based Measures in Assessing Spatial Accessibility to Mammography Service

We identified the locations of all 53 U.S. Food and Drug Administration (FDA)-accredited non-mobile mammography facilities during 1997–2001 in the study area from the FDA. The address of the facilities was geocoded to obtain latitude and longitude using ArcGIS (Version 9.3.1, ESRI inc., Redlands, CA). Based on three GIS approaches, we calculated nine measures of accessibility:

- nearest facility:
  - shortest travel time (DST),
  - average of first 5 shortest travel time (DST5);
- (iii) service density (DES); and
- Two-Step Floating Catchment Area (2SFCA) indices:
  - unweighted index (SAU),
  - continuous-weighted index (SAC),
  - 3-timezone-quick weighted index (SA3Q),
  - 3-timezone-slow weighted index (SA3S),
  - 6-timezone-quick weighted index (SA6Q),
  - 6-timezone-slow weighted index (SA6S).

We restricted the background population to women age 40 and above since screening mammography guidelines recommend mammography use for this population [23], [24].

#### A. Nearest facility (facilities)

We calculated the shortest travel time (DST) from the population-weighted centroid of each block group to mammography facilities using ArcGIS Network Analyst extension (Version 9.3.1, ESRI inc., Redlands, CA). We also calculated the average shortest travel time to the first five nearest facilities (DST5).

#### B. Service density

We calculated the service density (DES) by dividing the total number of mammography machines at the facilities that can be reached within 30 minutes (30-minute network buffer) from each block group centroid by this block group's population of women age 40+.
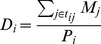
(1)Where 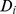 represents the density of block group 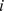; 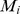 is the number of mammography machines at facility 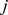; 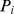 represents the women population age 40+ at block group 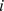; 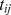 is travel time (zone) from census block group 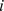 to mammography facility 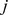 which can be reached with 30 minutes from the block group 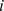.

#### C. Two-Step Floating Catchment Area (2SFCA) Method

We applied the 2SFCA method to compute a spatial accessibility score for each Census block group. First, we computed the network road travel time matrix between all mammography facilities and all Census block group population-weighted centroids using ArcGIS Network Analyst extension (Version 9.3.1, ESRI inc., Redlands, CA). Maximum catchment range was set to 30-minute travel time (driving) based on other accessibility studies [17], [18]. Second, we calculated the mammography machine-to-population (women population age 40 and above) of each mammography facility by dividing the number of machines by the weighted population of all Census block groups which centroids fall into the 30-minute catchment area of that facility (Equation 2).
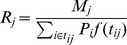
(2)Where 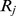 denotes the ratio of mammography machines to population for facility 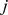, while 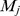 is the number of mammography machines at facility 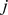; 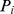 is the population of block group 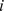; 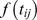 is the weighting function; and 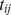 is travel time (zone) from census block group 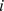 to mammography facility 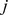.

Third, we calculated the spatial accessibility for each Census block group (Equation 3).
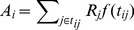
(3)Where 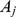 denotes the spatial accessibility of census block group 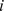.

We weighted the population and machine-to-population ratio using a zonal Gaussian decay function which was thought of as an appropriate weighting function regarding distance decay in the zonal-weighted 2SFCA models [25] (Equation 4) and a continuous Gaussian weighting function in the continuous weighted 2SFCA model [19] (Equation 5).
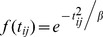
(4)

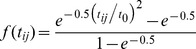
(5)Where 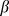 is the empirical parameter in the decay function; 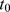 is the maximum catchment travel time (30 minutes in current study). In addition, we also used the original unweighted 2SFCA model in which 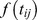 = 1.

Because it is unclear how the decay of the travel time affect our findings, we used 3 time zones (per 10-minute travel time) quick-decay (1.00, 0.51 and 0.07) and slow-decay (1.00, 0.75 and 0.32) and 6 time zones (per 5-minute travel time) quick-decay (1.00, 0.82, 0.45, 0.17, 0.04 and 0.01) and slow-decay function parameters (1.00, 0.96, 0.85, 0.70, 0.53 and 0.37) in the zonal-weighted 2SFCA models as part of a sensitivity analysis. We examined the locations of all mammography facilities and found a slight change in the number of mammography facilities over time. Nevertheless, to minimize the potential effect on our findings, we computed the spatial accessibility for each year and applied the 5-year average as the final spatial accessibility score.

### Neighborhood Socioeconomic Deprivation

It is well-known that women have lower screening mammography use in neighborhoods with more socioeconomic (SES) deprivation [26]. In this study, we regarded neighborhood socioeconomic deprivation as a potential neighborhood confounder. Referring to our previous study [27], we selected 21 Census variables from 2000 U.S. Census in six domains to construct a composite Census block group-level socioeconomic deprivation index using a multivariate approach. These domains included education, occupation, housing conditions, income and poverty, racial composition, and residential stability (Table 1). A common factor analysis with varimax rotation was applied to construct a deprivation factor from the 21 Census variables. Variables with significantly larger factor loading on the deprivation factor were selected to build the socioeconomic deprivation index and its internal consistency was evaluated using Cronbach's alpha coefficient.

**Table 1:** Characteristics of Census variables composing census block group socioeconomic (SES) deprivation index.

Domain	Census Variable	Factor Loading	Factor Scoring Coefficient
Education			
	% total population with less than high school	0.46353	0.05951
	% total population with a college degree	−0.42384	0.01817
Occupation			
	% civilian labor force unemployed*	0.79788	0.20320
	% White collar §	−0.46336	−0.00944
Housing			
	% household (HH) rent	0.58504	0.05888
	% vacant HH*	0.81785	0.16175
	% HH with > = 1 person per room*	0.61979	0.10684
	Median value of all owner-occupied HH, $	−0.19721	0.12412
	% female headed HH with dependent children† *	0.67542	0.10635
	% HH on public assistance income*	0.81928	0.17055
	% HH with no vehicle*	0.82504	0.15674
	% HH with no kitchen	0.10527	−0.12607
	% HH with no phone*	0.63371	0.08719
	% occupied HH with incomplete plumbing	0.20827	−0.07152
Income and Poverty			
	Median family income, $	−0.42799	0.06314
	% HH income> = 400% of the US median HH income	−0.03565	0.17685
	% population below federal poverty line*	0.86242	0.16229
Racial Composition			
	% non-Hispanic (NH) African Americans*	0.75122	0.15293
	% foreign born	−0.19410	−0.08588
Residential Stability			
	% persons in same house no less than 5 years	−0.25038	0.00338
	% residents aged 65 years and over	−0.15917	0.00100
	Proportion of total variance explained	44.1%	
	Cronbach's Alpha (internal consistency)	0.93	

§ White collar includes management, professional, and related occupations;

† % female headed HH with dependent children (no husband present with own children under 18 years;

* variables selected to compute the socioeconomic deprivation score.

### Statistical Analysis

To capture differences in the characteristics of the nine GIS-based measures, we performed the analyses in three aspects. First, we calculated Spearman rank correlation coefficients to compare their simple correlations. Second, we categorized all nine GIS-based measures into quartiles and computed weighted Kappa coefficients to examine their agreements. Quartiles reduce the effect of high and low prevalence on the Kappa coefficient [28]. The Kappa agreement was differentiated using a commonly cited scale: κ<0, no agreement; κ = 0.01–0.20, slight agreement; κ = 0.21–0.40, fair agreement; κ = 0.41–0.60, moderate agreement; κ = 0.61–0.80, substantial agreement; κ>0.80, perfect agreement [29]. Third, we computed global Moran's *I* indexes to compare differences in spatial autocorrelation, while we also performed Anselin local Moran's *I* tests to contrast their spatial patterns of these nine GIS-based measures. We specified the neighborhood relationship using the “Inverse Distance” weight function to obtain Moran's I statistics. All spatial features are assumed to impact on one another, but the farther away a feature is, the smaller influence it has [30]. The global Moran's *I* index is a spatial autocorrelation measure (feature similarity) ranging from −1 to 1. A value closer to 1 for Moran's *I* index suggests a more clustered global spatial pattern, while a value closer to −1 suggests a more dispersed global spatial pattern. A completely random spatial pattern exists when Moran's *I* is zero [30]. Anselin local Moran's I test is a tool to identify contiguous neighborhoods with values similar in magnitude (either high or low) and spatial outliers [30]. A spatial outlier indicates that a local region with high value is surrounded by neighborhoods with significantly low values, or vice versa.

As an indicator of predictive validity, we examined the associations of nine GIS-based measures with neighborhood risk of late-stage breast cancer. We applied a generalized linear mixed model to fit the multilevel logistic regression. All breast cancer cases were nested within their residential census block groups. The nine spatial accessibility measures and the socioeconomic deprivation index were dichotomized to below and above the median to facilitate interpretation. To examine the effect of spatial accessibility on late-stage breast cancer and the impact of neighborhood socioeconomic deprivation, we fitted the models in three ways. First, we used multivariate models that were adjusted for demographics and neighborhood socioeconomic deprivation to examine the independent effect of spatial accessibility. Second, we used jointly-classified models by combining the two categories of spatial accessibility and the two categories of neighborhood socioeconomic deprivation into one variable with four categories, which examines nonlinear effects of the combination of both variables. Third, we used stratified models in which the effect of spatial accessibility was examined in each stratum of neighborhood socioeconomic deprivation, which examines the interaction between both variables. Scaled deviance was used to evaluate the goodness-of-fit of model fitting with smaller value indicating a better fitting.

The data were managed and analyzed using SAS (Version 9.2, SAS Institute Inc., Cary, NC). Global and local Moran's *I* analyses were computed using ArcGIS spatial statistics tools package and GIS mapping were performed in ArcMap (ArcGIS, Version 9.3.1, ESRI Inc., Redlands, CA).

## Results

**Figure 1:**
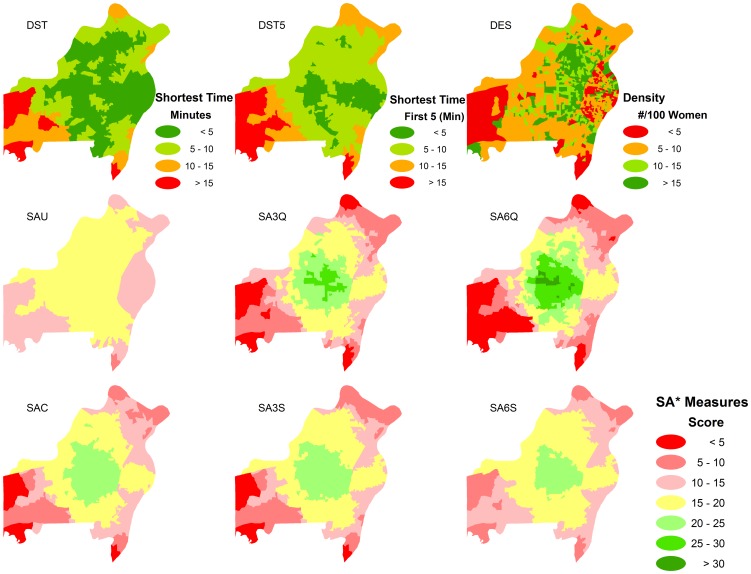
Distribution of nine GIS-based measures in access to mammography in St. Louis.

Service density measures had a much broader range than measures using shortest travel time(s) and 2SFCA methods (Table 2). The spatial pattern of neighborhood accessibility to mammography service using different spatial methods is shown in Figure 1. For the 2SFCA measures, the methods with distance decay weighting showed a larger variation and broader spatial accessibility ranges (Table 1 and Figure 1) compared to the un-weighted method (SAU vs. SAC, SA3Q, SA3S, SA6Q and SA6S), while quicker zonal-weighting made the SA structure broader than slower zonal-weighting (SA3Q vs. SA3S and SA6Q vs. SA6S).

**Table 2:** Distribution of nine GIS-based measures in St. Louis.

Variable	Mean	STD	Min	P25	Median	P75	Max	IQR	Range
Nearest facility									
DST^a^	4.31	2.41	0.23	2.61	3.87	5.44	18.30	2.84	18.06
DST5^b^	6.37	2.52	1.16	4.56	6.11	7.50	20.68	2.94	19.52
Service density									
DES^c^	12.70	11.37	1.76	6.52	9.79	15.96	236.00	9.44	234.24
Spatial accessibility									
SAU^d^	16.15	1.33	8.77	14.88	16.01	17.64	17.64	2.77	8.87
SAC^e^	16.39	3.20	3.42	14.67	15.45	18.96	23.26	4.30	19.84
SA3Q^f^	16.50	4.32	2.57	14.23	15.87	18.94	28.04	4.71	25.47
SA3S^g^	16.41	3.41	4.21	14.58	15.87	18.73	24.40	4.14	20.19
SA6Q^h^	16.63	5.65	1.10	13.46	15.66	19.92	33.61	6.45	32.51
SA6S^i^	16.31	2.49	5.51	14.81	15.42	18.57	21.40	3.76	15.89

a : shortest travel time (minutes);

b : average travel time to the nearest five facilities (minutes);

c : service density;

d : spatial accessibility index from the model without weighting parameter;

e : spatial accessibility index from the model with continuous weighting parameter;

f : spatial accessibility index from the zonal weighted model with 3 time zones and quick decay weighting;

g : spatial accessibility index from the zonal weighted model with 3 time zones and slow decay weighting;

h : spatial accessibility index from the zonal weighted model with 6 time zones and quick decay weighting;

i : spatial accessibility index from the zonal weighted model with 6 time zones and slow decay weighting.

The principal components common factor analysis identified the first common factor as the deprivation factor which explained 44.1% of the total variance. The nine Census variables, with large factor loading on the deprivation factor, included the percentage of civilian labor force unemployed, the percentage of vacant households, the percentage of households with no less than one person per room, the percentage of female headed households with dependent children, the percentage of households with public assistance income, the percentage of households with no vehicle, the percentage of households with no phone, the percentage of population below federal poverty line, and the percentage of non-Hispanic African Americans. These nine Census variables indicated a high internal consistency (Cronbach's alpha = 0.93, Table 1).

The distributions of nine GIS-based measures are skewed. Although the correlation of all GIS-based measures are statistically significant, Spearman rank correlation coefficients showed that measures of shortest travel time(s) have low correlations with service density measure 0.076< = rho< = 0.132) and slightly higher correlations with 2SFCA measures (0.087< = rho< = 0.580). Service density measures are moderately correlated with 2SFCA measures. 2SFCA measures are highly correlated (rho> = 0.606) as shown Table 3.

**Table 3:** Spearman correlations between nine GIS-based measures in access to mammography in St. Louis.

	DST	DST5	DES	SAU	SAC	SA3Q	SA3S	SA6Q	SA6S
DST	1.00	0.8448	0.1320	0.1633	−0.2431	−0.3160	−0.2291	−0.4060	−0.1678
DST5	-	1.00	0.0764	0.0867	−0.4083	−0.4846	−0.3889	−0.5797	−0.3183
DES	-	-	1.00	0.5064	0.3765	0.3351	0.3819	0.2857	0.4114
SAU	-	-	-	1.00	0.7751	0.6914	0.7782	0.6060	0.8302
SAC	-	-	-	-	1.00	0.9514	0.9703	0.9343	0.9769
SA3Q	-	-	-	-	-	1.00	0.9838	0.9722	0.9448
SA3S	-	-	-	-	-	-	1.00	0.9326	0.9807
SA6Q	-	-	-	-	-	-	-	1.00	0.8963
SA6S	-	-	-	-	-	-	-	-	1.00

(DST: shortest travel time; DST5: average of 5 shortest travel time; DES: density; SAU: spatial accessibility index from the model without weighting parameter; SAC: spatial accessibility index from the model with continuous weighting parameter; SA3Q: spatial accessibility index from the zonal weighted model with 3 time zones and quick decay weighting; SA3S: spatial accessibility index from the zonal weighted model with 3 time zones and slow decay weighting; SA6Q: spatial accessibility index from the zonal weighted model with 6 time zones and quick decay weighting; SA6S: spatial accessibility index from the zonal weighted model with 6 time zones and slow decay weighting.); all coefficients are statistically significant.

Table 4 showed the Kappa coefficients of nine GIS-based measures. Measures of shortest travel time (s) have no agreement with service density measure (κ<0), and slight agreement with 2SFCA measures (κ< = 0.23), while service density has slight or fair agreement with 2SFCA measures (κ<0.40). The 2SFCA measures have higher agreement with each other (κ = 0.48–0.90).

**Table 4:** Weighted Kappa (95% confidence intervals) for nine GIS-based measures of access to mammography in St. Louis.

	DST	DST5	DES	SAU	SAC	SA3Q	SA3S	SA6Q
DST	-	-	-	-	-	-	-	-
DST5	0.65 (0.62, 0.68)	-	-	-	-	-	-	-
DES	−0.11 (−0.15, −0.07)	−0.08 (−0.12, −0.04)	-	-	-	-	-	-
SAU	−0.11 (−0.15, −0.07)	−0.06 (−0.11, −0.02)	0.32 (0.28, 0.36)	-	-	-	-	-
SAC	0.10 (0.06, 0.15)	0.18 (0.13, 0.22)	0.26 (0.22, 0.30)	0.61 (0.58, 0.64)	-	-	-	-
SA3Q	0.15 (0.11, 0.20)	0.25 (0.21, 0.29)	0.24 (0.20, 0.29)	0.52 (0.49, 0.55)	0.82 (0.80, 0.84)	-	-	-
SA3S	0.10 (0.05, 0.14)	0.18 (0.14, 0.22)	0.26 (0.22, 0.30)	0.58 (0.55, 0.61)	0.84 (0.82, 0.86)	0.90 (0.88, 0.91)	-	-
SA6Q	0.23 (0.18, 0.27)	0.33 (0.29, 0.37)	0.19 (0.14, 0.23)	0.48 (0.44, 0.51)	0.79 (0.77, 0.82)	0.87 (0.85, 0.89)	0.78 (0.75, 0.80)	-
SA6S	0.03 (−0.01, 0.07)	0.10 (0.06, 0.14)	0.30 (0.26, 0.34)	0.66 (0.63, 0.68)	0.87 (0.86, 0.89)	0.81 (0.79, 0.83)	0.89 (0.88, 0.91)	0.71 (0.68, 0.74)

(DST: shortest travel time; DST5: average of 5 shortest travel time; DES: density; SAU: spatial accessibility index from the model without weighting parameter; SAC: spatial accessibility index from the model with continuous weighting parameter; SA3Q: spatial accessibility index from the zonal weighted model with 3 time zones and quick decay weighting; SA3S: spatial accessibility index from the zonal weighted model with 3 time zones and slow decay weighting; SA6Q: spatial accessibility index from the zonal weighted model with 6 time zones and quick decay weighting; SA6S: spatial accessibility index from the zonal weighted model with 6 time zones and slow decay weighting.)

**Figure 2:**
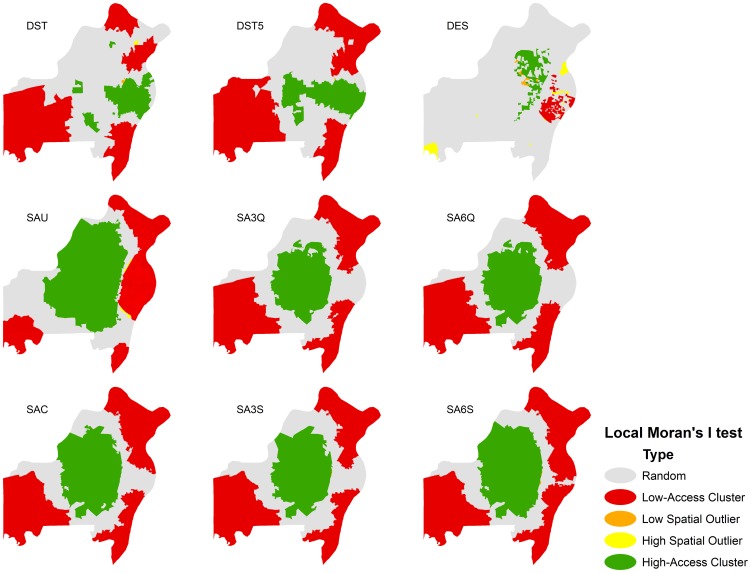
Spatial patterns (Anselin local Moran's I tests) of nine GIS-based measures in access to mammography in St. Louis.

Global Moran's *I* indicated that measures of shortest time(s) have medium spatial autocorrelation (Moran's *I* = 0.42 in DST and Moran's *I* = 0.48 in DST5), while service density has a low spatial autocorrelation (Moran's *I* = 0.12). Unweighted 2SFCA measure has a strong spatial autocorrelation (Moran's *I* = 0.71) and other 2SFCA measures have medium spatial autocorrelation (Moran's *I* = 0.43–0.50) (Table 5). Anselin local Moran's I tests exhibited considerable differences in spatial pattern of nine GIS-based measures (Figure 2). The area with high access based on shortest travel time(s) measures were located mainly in the east-central part of the study area, but in the study area's central part for the 2SFCA measures. The service density measures showed smaller cluster areas compared to other measures.

**Table 5:** Global Moran's I of nine GIS-based measures in access to mammography in St. Louis.

Variable	Moran's I (95% CI)
DST	0.42 (0.41–0.42)
DST5	0.48 (0.47–0.49)
DES	0.12 (0.11–0.13)
SAU	0.71 (0.71–0.72)
SAC	0.47 (0.46–0.47)
SA3Q	0.43 (0.43–0.44)
SA3S	0.45 (0.44–0.45)
SA6Q	0.44 (0.43–0.45)
SA6S	0.50 (0.49–0.51)

(DST: shortest travel time; DST5: average of 5 shortest travel time; DES: density; SAU: spatial accessibility index from the model without weighting parameter; SAC: spatial accessibility index from the model with continuous weighting parameter; SA3Q: spatial accessibility index from the zonal weighted model with 3 time zones and quick decay weighting; SA3S: spatial accessibility index from the zonal weighted model with 3 time zones and slow decay weighting; SA6Q: spatial accessibility index from the zonal weighted model with 6 time zones and quick decay weighting; SA6S: spatial accessibility index from the zonal weighted model with 6 time zones and slow decay weighting.)

Table 6 shows the effects of the nine spatial accessibility measures on the odds of late-stage breast cancer. The measure using the shortest travel time (multivariate odds ratio [OR] = 0.99, 95% confidence interval [CI]: 0.86–1.14), average of first five shortest travel time (multivariate OR = 0.95, 95% CI: 0.83–1.09), and service density (multivariate OR = 0.90, 95% CI: 0.79–1.04) were not associated with late-stage breast cancer. However, lower values on the spatial accessibility indices using the 6-timezone weighted methods were significantly associated with increased odds of late-stage breast cancer (SA6Q, age-race-adjusted OR: 1.16, 95% CI: 1.01–1.32; SA6S, age-race-adjusted OR: 1.21, 95% CI: 1.05–1.39). In the multivariable model, spatial accessibility index remained associated with late-stage breast cancer (SA6Q, OR: 1.15, 05% CI: 1.01–1.32; SA6S, OR: 1.19, 95% CI: 1.03–1.37). The effect of neighborhood socioeconomic deprivation disappeared after controlling for age, race and neighborhood spatial accessibility (more deprived vs. less deprived: age-adjusted OR: 1.31, 95% CI: 1.13–1.51; age-race-adjusted OR: 1.19, 95% CI: 1.00–1.42; multivariate-adjusted OR: 1.15, 95% CI: 0.96–1.37).

**Table 6:** Effects of block group spatial accessibility to mammography service and socioeconomic (SES) deprivation on risk of late-stage breast cancer.

	Odds Ratio (95% CI)*		
	Model I	Model II	Model III
CST^a^	0.97 (0.84 to 1.11)	0.97 (0.85 to 1.11)	0.99 (0.86 to 1.14)
SES	-	-	1.19 (1.00 to 1.42)
CST5^b^	0.92 (0.80 to 1.05)	0.93 (0.81 to 1.07)	0.95 (0.83 to 1.09)
SES	-	-	1.18 (0.99 to 1.41)
DEN^c^	0.89 (0.77 to 1.02)	0.90 (0.78 to 1.03)	0.90 (0.79 to 1.04)
SES	-	-	1.19 (1.00 to 1.42)
SAU^d^	1.23 (1.07 to 1.41)	1.15 (1.00 to 1.33)	1.12 (0.96 to 1.30)
SES	-	-	1.15 (0.96 to 1.38)
SAC^e^	1.16 (1.01 to 1.33)	1.12 (0.98 to 1.28)	1.11 (0.97 to 1.27)
SES	-	-	1.18 (0.99 to 1.41)
SA3Q^f^	1.13 (0.99 to 1.30)	1.09 (0.96 to 1.25)	1.09 (0.95 to 1.25)
SES	-	-	1.19 (1.00 to 1.42)
SA3S^g^	1.14 (1.00 to 1.31)	1.10 (0.96 to 1.26)	1.08 (0.95 to 1.24)
SES	-	–	1.18 (0.99 to 1.41)
SA6Q^h^	1.19 (1.04 to 1.36)	1.16 (1.01 to 1.32)	1.15 (1.01 to 1.32)
SES	-	-	1.19 (1.00 to 1.42)
SA6S^i^	1.26 (1.10 to 1.45)	1.21 (1.05 to 1.39)	1.19 (1.03 to 1.37)
SES	-	-	1.15 (0.96 to 1.37)
SES	1.31 (1.13 to 1.51)	1.19 (1.00 to 1.42)	-

Model I was adjusted for age only; Model II was adjusted for age and race; Model III included spatial accessibility score, socioeconomic score, age and race.

* Higher spatial accessibility and less deprivation were set as references;

a : shortest travel time (minutes);

b : average travel time to the nearest five facilities (minutes);

c : service density;

d : spatial accessibility index from the model without weighting parameter;

e : spatial accessibility index from the model with continuous weighting parameter;

f : spatial accessibility index from the zonal weighted model with 3 time zones and quick decay weighting;

g : spatial accessibility index from the zonal weighted model with 3 time zones and slow decay weighting;

h : spatial accessibility index from the zonal weighted model with 6 time zones and quick decay weighting;

i : spatial accessibility index from the zonal weighted model with 6 time zones and slow decay weighting.

Table 7 shows the combined effects of spatial accessibility and neighborhood socioeconomic deprivation on late-stage breast cancer. The odds of late-stage breast cancer in neighborhoods with lower spatial accessibility to mammography service and more socioeconomic deprivation was elevated (SA6Q, OR: 1.34, 95% CI: 1.07–1.69; SA6S, OR: 1.32, 95% CI: 1.07–1.64). The stratified models show that lower spatial accessibility to mammography service was associated with greater odds of late-stage breast cancer in less deprived neighborhoods (SA6Q, OR: 1.19, 95% CI: 1.02–1.40; SA6S, OR: 1.27, 95% CI: 1.07–1.50), but not in more deprived neighborhoods (SA6Q, OR: 1.06, 95% CI: 0.84–1.35; SA6S, OR: 1.04, 95% CI: 0.81–1.32).

**Table 7:** Combined effect of block group spatial accessibility to mammography service and socioeconomic (SES) deprivation on risk of late-stage breast cancer.

			Odds Ratio (95% CI)	
	Socioeconomic Condition	SA measures	Joint-Classified	Stratified
Model 1: CST^a^				
	Less deprived			
		More accessible	1.00	1.00
		Less accessible	1.03 (0.87 to 1.22)	1.03 (0.87 to 1.22)
	More deprived			
		More accessible	1.26 (0.99 to 1.60)	1.00
		Less accessible	1.17 (0.91 to 1.49)	0.92 (0.73 to 1.17)
Model 2: CST5^b^				
	Less deprived			
		More accessible	1.00	1.00
		Less accessible	0.97 (0.82 to 1.15)	0.97 (0.82 to 1.15)
	More deprived			
		More accessible	1.23 (0.97 to 1.56)	1.00
		Less accessible	1.11 (0.87 to 1.42)	0.91 (0.72 to 1.15)
Model 3: DEN^c^				
	Less deprived			
		More accessible	1.00	1.00
		Less accessible	0.82 (0.69 to 0.97)	0.82 (0.69 to 0.97)
	More deprived			
		More accessible	0.99 (0.78 to 1.27)	1.00
		Less accessible	1.10 (0.88 to 1.38)	1.11 (0.87 to 1.41)
Model 4: SAU^d^				
	Less deprived			
		More accessible	1.00	1.00
		Less accessible	1.15 (0.96 to 1.39)	1.15 (0.96 to 1.39)
	More deprived			
		More accessible	1.20 (0.95 to 1.51)	1.00
		Less accessible	1.27 (1.03 to 1.57)	1.06 (0.83 to 1.36)
Model 5: SAC^e^				
	Less deprived			
		More accessible	1.00	1.00
		Less accessible	1.15 (0.97 to 1.36)	1.15 (0.97 to 1.36)
	More deprived			
		More accessible	1.25 (0.99 to 1.57)	1.00
		Less accessible	1.27 (1.02 to 1.59)	1.02 (0.80 to 1.30)
Model 6: SA3Q^f^				
	Less deprived			
		More accessible	1.00	1.00
		Less accessible	1.15 (0.98 to 1.35)	1.15 (0.98 to 1.35)
	More deprived			
		More accessible	1.30 (1.03 to 1.64)	1.00
		Less accessible	1.26 (1.00 to 1.57)	0.96 (0.76 to 1.23)
Model 7: SA3S^g^				
	Less deprived			
		More accessible	1.00	1.00
		Less accessible	1.17 (0.99 to 1.37)	1.17 (0.99 to 1.37)
	More deprived			
		More accessible	1.32 (1.05 to 1.65)	1.00
		Less accessible	1.23 (0.98 to 1.53)	0.93 (0.73 to 1.18)
Model 8: SA6Q^h^				
	Less deprived			
		More accessible	1.00	1.00
		Less accessible	1.19 (1.02 to 1.40)	1.19 (1.02 to 1.40)
	More deprived			
		More accessible	1.26 (1.00 to 1.59)	1.00
		Less accessible	1.34 (1.07 to 1.69)	1.06 (0.84 to 1.35)
Model 9: SA6S^i^				
	Less deprived			
		More accessible	1.00	1.00
		Less accessible	1.27 (1.07 to 1.50)	1.27 (1.07 to 1.50)
	More deprived			
		More accessible	1.27 (1.01 to 1.61)	1.00
		Less accessible	1.32 (1.07 to 1.64)	1.04 (0.81 to 1.32)

All models were adjusted for age and race; “more accessible” means shorter travel time and bigger score values in density and 2SFCA measures, while “less accessible” means longer travel time and smaller score values in density and 2SFCA measures.

a : shortest travel time (minutes);

b : average travel time to the nearest five facilities (minutes);

c : service density;

d : spatial accessibility index from the model without weighting parameter;

e : spatial accessibility index from the model with continuous weighting parameter;

f : spatial accessibility index from the zonal weighted model with 3 time zones and quick decay weighting;

g : spatial accessibility index from the zonal weighted model with 3 time zones and slow decay weighting;

h : spatial accessibility index from the zonal weighted model with 6 time zones and quick decay weighting;

i : spatial accessibility index from the zonal weighted model with 6 time zones and slow decay weighting.

## Discussion

Our main purpose was to compare varied GIS-based measures of access to mammography service computed using three different spatial approaches, and we also determined the predictive validity in their association with odds of late-stage breast cancer. Our study demonstrated that the correlation and agreement among the different measures (shortest travel time, service density and 2SFCA measures) was low. Also, the spatial pattern of the measures varied considerably. Only measures using the 6-timezone-weighted 2SFCA method were significantly associated with increased neighborhood odds of late-stage breast cancer after accounting for demographics and neighborhood socioeconomic deprivation. The effect of neighborhood socioeconomic deprivation could be explained in part by neighborhood spatial accessibility. Combined with more deprived neighborhood socioeconomic condition, lower spatial accessibility to mammography service is associated with greater neighborhood risk of late-stage breast cancer.

Service availability or density is the most common measure in assessing spatial accessibility due to its easy computation [3], [4], [5], [31], [32], [33], [34], [35], [36], [37], [38], [39], [40], [41], [42], [43], [44], [45], [46], [47], [48]. It does not require advanced GIS skills, and only need to link each service location to its corresponding neighborhood or a predefined buffered area in which that service facility is located. With the rapid development of GIS techniques, it also becomes convenient to compute the network-based distance based on a GIS road network layer. This results in the frequently used measure of the shortest travel time (or nearest travel distance) to the service locations for assessing service accessibility [6], [7], [10], [11], [12], [14], [49], [50], [51], [52], [53], [54], [55], [56], [57], [58], [59], [60], [61], [62], [63], [64], [65], [66], [67]. Recently, it has become feasible to create a composite accessibility index using a state-of-the-art two-Step Floating Catchment Area (2SFCA) approach. This approach is more reasonable than availability/density and nearest travel distance/shortest travel time through the integration of travel barrier and service competition, however, it is a more sophisticated technique requiring several sequential steps: first, one needs to compute a travel distance/time matrix between service location and population locations within a predefined catchment area using a GIS, such as ArcGIS Network Analyst. If a study involves large numbers of study neighborhoods and participant locations, this process could be very time-consuming. Second, one needs to compute the population-service ratio for each service location and then a composite accessibility score for each population location using statistical derivations with varied weighting techniques, such as the Enhanced 2SFCA method [18] and the Gaussian 2SFCA method [19]. Additionally, some efforts to improve the technical precision of the 2SFCA method make this approach more complex. Luo and Whippo explored an approach to reduce the bias due to the rural-urban difference through using predefined base population threshold and service-to-population ratio threshold to create varied catchment sizes for each service location and population location instead of fixed catchment size (fixed travel time or distance) [20]. Recently, Bell and Bissonnette et al developed an extension of the 2SFCA method, called the 3SFCA, aggregating the small-area spatial accessibility score to a larger study neighborhood [21], [68]. Both methods could substantially improve the measurement precision and reduce the influence of the rural-urban difference especially when the study area is large or irregular, meanwhile, they also add considerable computational burden if the study sample size is large. In the former approach, the travel distance/time matrix need to be computed under a much larger range, such as 30-minute travel, even 60- or 90-minute travel time, to capture the specific catchment sizes of each service and population location, while the latter one requires an additional step to obtain the accessibility measure.

Briefly, for most researchers, service availability/density and nearest travel distance/shortest travel time are easier to compute despite the fact that travel barriers or service competition is ignored. In contrast, the 2SFCA and its extended methods are more technical and require stronger computation skill to perform although this approach has methodological advantages. Therefore, it is necessary to compare these GIS-measures in principle and predictive validity for a specific study outcome. If no significant difference, service availability/density and/or nearest travel distance/shortest travel time could be applied instead of more complex 2SFCA approaches. Otherwise, it may be a better way to apply more advanced 2SFCA approach. It is noteworthy that, for the 2SFCA approach, the number of time zones and decay weighting parameters should be evaluated for different study outcomes. In our study, more time zones worked better while decay did not seem to play a role. In addition, for a study with large mixed area characteristics, rural-urban difference, such as different catchment sizes, may be considered when assessing spatial accessibility, including the application of varied catchment sizes [20] or the aggregation of small-area accessibility measures to larger neighborhoods [21].

Our study indicated that the GIS-based measures of spatial accessibility exhibit different characteristics. The findings suggest that the weighted 2SFCA method is better than service density and shortest travel time when assessing spatial accessibility to mammography service. Future studies should further investigate and improve the 2SFCA methods and compare GIS-based measures with perceived accessibility when assessing neighborhood effect of the distribution of mammography service. Appropriate assessment could reduce bias when investigating the effect of spatial accessibility on breast cancer outcomes. Additionally, precise and reliable measures of spatial accessibility to mammography cannot only provide justification for effective multilevel interventions, but also help local and state policy makers and health service planners identify service shortage areas to mammography and improve the allocation of mammography services to reduce geographic disparity in breast cancer-related outcomes that appears to exist in community settings. The selection of GIS-based measures can be extended to other areas of public health, including accessibility to other medical services, the food environment, and alcohol or cigarettes sale environments [33], [43], [48], [57], [62], [66].

There are several strengths to our study. We computed nine GIS-based measures of access to mammography services using three different spatial approaches, including shortest travel time, service density and the 2SFCA method, and systematically compared their correlation, agreement and spatial pattern within a single study region and population. The 2SFCA approach with more time zone-weighting appears to capture more details in spatial pattern and significant or stronger association of spatial accessibility to mammography service with late-stage breast cancer. We applied the number of mammography machines as the service capacity and the population of women age 40 and above as the screening-eligible population. We also used the Census block group as the geographic unit which is much smaller than Zip code and can lead to a more precise measurement of accessibility.

Our study also has some limitations. First, our findings may only be generalized to a metropolitan area. Results may be different when examining more rural areas [18]. Second, the estimation of spatial accessibility for block groups at the edge of the study area boundary could have been underestimated since we did not include facilities outside the study area. However, this is unlikely to have affected our findings because there was only one mammography facility near the Missouri river. On the west-side and east-side of the study area, the Missouri river and Mississippi river formed a natural boundary. Third, except for age and race, our study did not include other individual-level factors that are associated with late-stage breast cancer, such as marital status, low education, unemployment, health insurance coverage, non-participation in regular general health check-up, low interest in health issues and diagnostic delay [69], [70], [71], [72], [73]. Additionally, our study assumed that all women with the same travel time had equal opportunity to access a mammography facility, that all facilities had similar quality of provided services, and that each mammography radiologist in each mammography facility had equal capacity to read mammography films. Women with lower income or without health insurance coverage might seek mammography service from safety net providers even if these locations might be farther and have lower quality services than other facilities. Regardless of these limitations, our findings provide helpful information to policy makers about where accessibility to needed mammography services is lower and counteract this in order to reduce the odds of late-stage breast cancer diagnosis. Future studies could include additional risk factors and service facility characteristics to validate the independent effect of spatial accessibility to mammography service.

In conclusion, different GIS-based measures appear to describe different concepts based on their intercorrelations, agreements and spatial patterns. Caution should be exercised in selecting a spatial approach in assessing access to mammography when investigating neighborhood contextual effects on breast cancer outcomes. The 2SFCA measure appears to be the best approach based on theoretical considerations, spatial patterns and predictive validity. Our findings suggest that the 2SFCA approach can be a valuable option for epidemiologists when investigating the health effects of the distributions of regional accessibility to services.

## References

[1] Jemal A, Siegel R, Xu J, Ward E (2010) Cancer statistics, 2010. CA Cancer J Clin 60: 277–300. 20610543 10.3322/caac.20073

[2] Berry DA, Cronin KA, Plevritis SK, Fryback DG, Clarke L, et al (2005) Effect of screening and adjuvant therapy on mortality from breast cancer. N Engl J Med 353: 1784–1792. 16251534 10.1056/NEJMoa050518

[3] Marchick J, Henson DE (2005) Correlations between access to mammography and breast cancer stage at diagnosis. Cancer 103: 1571–1580. 15772962 10.1002/cncr.20915

[4] Elting LS, Cooksley CD, Bekele BN, Giordano SH, Shih YC, et al (2009) Mammography capacity impact on screening rates and breast cancer stage at diagnosis. Am J Prev Med 37: 102–108. 19524392 10.1016/j.amepre.2009.03.017

[5] Elkin EB, Ishill NM, Snow JG, Panageas KS, Bach PB, et al (2010) Geographic access and the use of screening mammography. Med Care 48: 349–356. 20195174 10.1097/MLR.0b013e3181ca3ecb PMC3647348

[6] Meersman SC, Breen N, Pickle LW, Meissner HI, Simon P (2009) Access to mammography screening in a large urban population: a multi-level analysis. Cancer Causes Control 20: 1469–1482. 19543987 10.1007/s10552-009-9373-4 PMC2746895

[7] Hyndman JC, Holman CD, Dawes VP (2000) Effect of distance and social disadvantage on the response to invitations to attend mammography screening. J Med Screen 7: 141–145. 11126163 10.1136/jms.7.3.141

[8] Gumpertz ML, Pickle LW, Miller BA, Bell BS (2006) Geographic patterns of advanced breast cancer in Los Angeles: associations with biological and sociodemographic factors (United States). Cancer Causes Control 17: 325–339. 16489540 10.1007/s10552-005-0513-1

[9] Mobley LR, Kuo TM, Clayton LJ, Evans WD (2009) Mammography facilities are accessible, so why is utilization so low? Cancer Causes Control 20: 1017–1028. 19205911 10.1007/s10552-009-9295-1 PMC2694850

[10] Engelman KK, Hawley DB, Gazaway R, Mosier MC, Ahluwalia JS, et al (2002) Impact of geographic barriers on the utilization of mammograms by older rural women. J Am Geriatr Soc 50: 62–68. 12028248 10.1046/j.1532-5415.2002.50009.x

[11] Celaya MO, Berke EM, Onega TL, Gui J, Riddle BL, et al (2010) Breast cancer stage at diagnosis and geographic access to mammography screening (New Hampshire, 1998–2004). Rural Remote Health 10: 1361. 20438282 PMC5585775

[12] Zenk SN, Tarlov E, Sun J (2006) Spatial equity in facilities providing low- or no-fee screening mammography in Chicago neighborhoods. J Urban Health 83: 195–210. 16736369 10.1007/s11524-005-9023-4 PMC2527168

[13] McLafferty S, Wang F (2009) Rural reversal? Rural-urban disparities in late-stage cancer risk in Illinois. Cancer 115: 2755–2764. 19434667 10.1002/cncr.24306 PMC2774239

[14] Peipins LA, Graham S, Young R, Lewis B, Foster S, et al (2011) Time and Distance Barriers to Mammography Facilities in the Atlanta Metropolitan Area. J Community Health 10.1007/s10900-011-9359-5 PMC5836475 21267639

[15] Yang DH, Goerge R, Mullner R (2006) Comparing GIS-based methods of measuring spatial accessibility to health services. J Med Syst 30: 23–32. 16548411 10.1007/s10916-006-7400-5

[16] Guagliardo MF (2004) Spatial accessibility of primary care: concepts, methods and challenges. Int J Health Geogr 3: 3. 14987337 10.1186/1476-072X-3-3 PMC394340

[17] Wang F, Luo W (2005) Assessing spatial and nonspatial factors for healthcare access: towards an integrated approach to defining health professional shortage areas. Health Place 11: 131–146. 15629681 10.1016/j.healthplace.2004.02.003

[18] Luo W, Qi Y (2009) An enhanced two-step floating catchment area (E2SFCA) method for measuring spatial accessibility to primary care physicians. Health Place 15: 1100–1107. 19576837 10.1016/j.healthplace.2009.06.002

[19] Dai D (2010) Black residential segregation, disparities in spatial access to health care facilities, and late-stage breast cancer diagnosis in metropolitan Detroit. Health Place 16: 1038–1052. 20630792 10.1016/j.healthplace.2010.06.012

[20] Luo W, Whippo T (2012) Variable catchment sizes for the two-step floating catchment area (2SFCA) method. Health Place 18: 789–795. 22560115 10.1016/j.healthplace.2012.04.002

[21] Bissonnette L, Wilson K, Bell S, Shah TI (2012) Neighbourhoods and potential access to health care: The role of spatial and aspatial factors. Health Place 18: 841–853. 22503565 10.1016/j.healthplace.2012.03.007

[22] Schootman M, Jeffe DB, Gillanders WE, Yan Y, Jenkins B, et al (2007) Geographic clustering of adequate diagnostic follow-up after abnormal screening results for breast cancer among low-income women in Missouri. Ann Epidemiol 17: 704–712. 17574437 10.1016/j.annepidem.2007.03.017

[23] US Preventive Services Task Force (2009) Screening for breast cancer: U.S. Preventive Services Task Force recommendation statement. Ann Intern Med 151: 716–726, W-236. 19920272 10.7326/0003-4819-151-10-200911170-00008

[24] Smith RA, Cokkinides V, Brooks D, Saslow D, Brawley OW (2010) Cancer screening in the United States, 2010: a review of current American Cancer Society guidelines and issues in cancer screening. CA Cancer J Clin 60: 99–119. 20228384 10.3322/caac.20063

[25] Wang L (2007) Immigration, ethnicity, and accessibility to culturally diverse family physicians. Health Place 13: 656–671. 17174590 10.1016/j.healthplace.2006.10.001

[26] Schootman M, Jeffe DB, Baker EA, Walker MS (2006) Effect of area poverty rate on cancer screening across US communities. J Epidemiol Community Health 60: 202–207. 16476748 10.1136/jech.2005.041020 PMC2465556

[27] Lian M, Schootman M, Doubeni CA, Park Y, Major JM, et al (2011) Geographic Variation in Colorectal Cancer Survival and the Role of Small-Area Socioeconomic Deprivation: A Multilevel Survival Analysis of the NIH-AARP Diet and Health Study Cohort. Am J Epidemiol 174: 828–838. 21836166 10.1093/aje/kwr162 PMC3203377

[28] Feinstein AR, Cicchetti DV (1990) High agreement but low kappa: I. The problems of two paradoxes. J Clin Epidemiol 43: 543–549. 2348207 10.1016/0895-4356(90)90158-l

[29] Landis JR, Koch GG (1977) The measurement of observer agreement for categorical data. Biometrics 33: 159–174. 843571

[30] ArcGIS Desktop Help (2009) Version 9.3.1. Environmental Systems Research Institute, Inc. (ESRI), Redlands, CA.

[31] Scribner RA, Cohen DA, Farley TA (1998) A geographic relation between alcohol availability and gonorrhea rates. Sex Transm Dis 25: 544–548. 9858351 10.1097/00007435-199811000-00009

[32] Cummins SC, McKay L, MacIntyre S (2005) McDonald's restaurants and neighborhood deprivation in Scotland and England. Am J Prev Med 29: 308–310. 16242594 10.1016/j.amepre.2005.06.011

[33] Cohen DA, Ghosh-Dastidar B, Scribner R, Miu A, Scott M, et al (2006) Alcohol outlets, gonorrhea, and the Los Angeles civil unrest: a longitudinal analysis. Soc Sci Med 62: 3062–3071. 16423436 10.1016/j.socscimed.2005.11.060 PMC2040035

[34] Moore LV, Diez Roux AV (2006) Associations of neighborhood characteristics with the location and type of food stores. Am J Public Health 96: 325–331. 16380567 10.2105/AJPH.2004.058040 PMC1470485

[35] Teach SJ, Guagliardo MF, Crain EF, McCarter RJ, Quint DM, et al (2006) Spatial accessibility of primary care pediatric services in an urban environment: association with asthma management and outcome. Pediatrics 117: S78–85. 16777835 10.1542/peds.2005-2000E

[36] Diez Roux AV, Evenson KR, McGinn AP, Brown DG, Moore L, et al (2007) Availability of recreational resources and physical activity in adults. Am J Public Health 97: 493–499. 17267710 10.2105/AJPH.2006.087734 PMC1805019

[37] Scribner R, Theall KP, Ghosh-Dastidar B, Mason K, Cohen D, et al (2007) Determinants of social capital indicators at the neighborhood level: a longitudinal analysis of loss of off-sale alcohol outlets and voting. J Stud Alcohol Drugs 68: 934–943. 17960312 10.15288/jsad.2007.68.934

[38] Bluthenthal RN, Cohen DA, Farley TA, Scribner R, Beighley C, et al (2008) Alcohol availability and neighborhood characteristics in Los Angeles, California and southern Louisiana. J Urban Health 85: 191–205. 18228148 10.1007/s11524-008-9255-1 PMC2430119

[39] Moore LV, Diez Roux AV, Brines S (2008) Comparing Perception-Based and Geographic Information System (GIS)-based characterizations of the local food environment. J Urban Health 85: 206–216. 18247121 10.1007/s11524-008-9259-x PMC2430123

[40] Moore LV, Diez Roux AV, Evenson KR, McGinn AP, Brines SJ (2008) Availability of recreational resources in minority and low socioeconomic status areas. Am J Prev Med 34: 16–22. 18083446 10.1016/j.amepre.2007.09.021 PMC2254179

[41] Schonlau M, Scribner R, Farley TA, Theall K, Bluthenthal RN, et al (2008) Alcohol outlet density and alcohol consumption in Los Angeles county and southern Louisiana. Geospat Health 3: 91–101. 19021112 10.4081/gh.2008.235 PMC3741099

[42] Campbell CA, Hahn RA, Elder R, Brewer R, Chattopadhyay S, et al (2009) The effectiveness of limiting alcohol outlet density as a means of reducing excessive alcohol consumption and alcohol-related harms. Am J Prev Med 37: 556–569. 19944925 10.1016/j.amepre.2009.09.028

[43] Fu LY, Cowan N, McLaren R, Engstrom R, Teach SJ (2009) Spatial accessibility to providers and vaccination compliance among children with medicaid. Pediatrics 124: 1579–1586. 19933734 10.1542/peds.2009-0233

[44] Maroko AR, Maantay JA, Sohler NL, Grady KL, Arno PS (2009) The complexities of measuring access to parks and physical activity sites in New York City: a quantitative and qualitative approach. Int J Health Geogr 8: 34. 19545430 10.1186/1476-072X-8-34 PMC2708147

[45] Theall KP, Scribner R, Ghosh-Dastidar B, Cohen D, Mason K, et al (2009) Neighbourhood alcohol availability and gonorrhea rates: impact of social capital. Geospat Health 3: 241–255. 19440966 10.4081/gh.2009.224 PMC6167931

[46] Kwate NO, Loh JM (2010) Separate and unequal: the influence of neighborhood and school characteristics on spatial proximity between fast food and schools. Prev Med 51: 153–156. 20457178 10.1016/j.ypmed.2010.04.020

[47] Cerin E, Frank LD, Sallis JF, Saelens BE, Conway TL, et al (2011) From neighborhood design and food options to residents' weight status. Appetite 56: 693–703. 21335040 10.1016/j.appet.2011.02.006

[48] Jennings A, Welch A, Jones AP, Harrison F, Bentham G, et al (2011) Local food outlets, weight status, and dietary intake: associations in children aged 9–10 years. Am J Prev Med 40: 405–410. 21406273 10.1016/j.amepre.2010.12.014 PMC3773911

[49] Athas WF, Adams-Cameron M, Hunt WC, Amir-Fazli A, Key CR (2000) Travel distance to radiation therapy and receipt of radiotherapy following breast-conserving surgery. J Natl Cancer Inst 92: 269–271. 10655446 10.1093/jnci/92.3.269

[50] Nattinger AB, Kneusel RT, Hoffmann RG, Gilligan MA (2001) Relationship of distance from a radiotherapy facility and initial breast cancer treatment. J Natl Cancer Inst 93: 1344–1346. 11535710 10.1093/jnci/93.17.1344

[51] Lovett A, Haynes R, Sunnenberg G, Gale S (2002) Car travel time and accessibility by bus to general practitioner services: a study using patient registers and GIS. Soc Sci Med 55: 97–111. 12137192 10.1016/s0277-9536(01)00212-x

[52] Meden T, St John-Larkin C, Hermes D, Sommerschield S (2002) MSJAMA. Relationship between travel distance and utilization of breast cancer treatment in rural northern Michigan. Jama 287: 111. 11754721

[53] Zenk SN, Schulz AJ, Israel BA, James SA, Bao S, et al (2005) Neighborhood racial composition, neighborhood poverty, and the spatial accessibility of supermarkets in metropolitan Detroit. Am J Public Health 95: 660–667. 15798127 10.2105/AJPH.2004.042150 PMC1449238

[54] Celaya MO, Rees JR, Gibson JJ, Riddle BL, Greenberg ER (2006) Travel distance and season of diagnosis affect treatment choices for women with early-stage breast cancer in a predominantly rural population (United States). Cancer Causes Control 17: 851–856. 16783613 10.1007/s10552-006-0025-7

[55] Burns CM, Inglis AD (2007) Measuring food access in Melbourne: access to healthy and fast foods by car, bus and foot in an urban municipality in Melbourne. Health Place 13: 877–885. 17470408 10.1016/j.healthplace.2007.02.005

[56] Pearce J, Blakely T, Witten K, Bartie P (2007) Neighborhood deprivation and access to fast-food retailing: a national study. Am J Prev Med 32: 375–382. 17478262 10.1016/j.amepre.2007.01.009

[57] Pearce J, Hiscock R, Blakely T, Witten K (2008) The contextual effects of neighbourhood access to supermarkets and convenience stores on individual fruit and vegetable consumption. J Epidemiol Community Health 62: 198–201. 18272733 10.1136/jech.2006.059196

[58] Pearce J, Mason K, Hiscock R, Day P (2008) A national study of neighbourhood access to gambling opportunities and individual gambling behaviour. J Epidemiol Community Health 62: 862–868. 18791042 10.1136/jech.2007.068114

[59] Sharkey JR, Horel S (2008) Neighborhood socioeconomic deprivation and minority composition are associated with better potential spatial access to the ground-truthed food environment in a large rural area. J Nutr 138: 620–627. 18287376 10.1093/jn/138.3.620

[60] Witten K, Hiscock R, Pearce J, Blakely T (2008) Neighbourhood access to open spaces and the physical activity of residents: a national study. Prev Med 47: 299–303. 18533242 10.1016/j.ypmed.2008.04.010

[61] Zenk SN, Powell LM (2008) US secondary schools and food outlets. Health Place 14: 336–346. 17881277 10.1016/j.healthplace.2007.08.003

[62] Pearce J, Hiscock R, Moon G, Barnett R (2009) The neighbourhood effects of geographical access to tobacco retailers on individual smoking behaviour. J Epidemiol Community Health 63: 69–77. 18628269 10.1136/jech.2007.070656

[63] Malqvist M, Sohel N, Do TT, Eriksson L, Persson LA (2010) Distance decay in delivery care utilisation associated with neonatal mortality. A case referent study in northern Vietnam. BMC Public Health 10: 762. 21144058 10.1186/1471-2458-10-762 PMC3009650

[64] Smith DM, Cummins S, Taylor M, Dawson J, Marshall D, et al (2010) Neighbourhood food environment and area deprivation: spatial accessibility to grocery stores selling fresh fruit and vegetables in urban and rural settings. Int J Epidemiol 39: 277–284. 19491142 10.1093/ije/dyp221

[65] Wong LY, Heng BH, Cheah JT, Tan CB (2010) Using spatial accessibility to identify polyclinic service gaps and volume of under-served population in Singapore using Geographic Information System. Int J Health Plann Manage 10.1002/hpm.1063 20672252

[66] Astell-Burt T, Flowerdew R, Boyle PJ, Dillon JF (2011) Does geographic access to primary healthcare influence the detection of hepatitis C? Soc Sci Med 10.1016/j.socscimed.2011.02.015 21481509

[67] Gatrell AC, Wood DJ (2012) Variation in geographic access to specialist inpatient hospices in England and Wales. Health Place 18: 832–840. 22522100 10.1016/j.healthplace.2012.03.009

[68] Bell S, Wilson K, Bissonnette L, Shah TI (in press) Access to primary health care: does neighbourhood of residence matter? Annal of the Association of American Geographers

[69] Nayeri K, Pitaro G, Feldman JG (1992) Marital status and stage at diagnosis in cancer. N Y State J Med 92: 8–11. 1574233

[70] Catalano RA, Satariano WA (1998) Unemployment and the likelihood of detecting early-stage breast cancer. Am J Public Health 88: 586–589. 9550999 10.2105/ajph.88.4.586 PMC1508418

[71] Ayanian JZ, Kohler BA, Abe T, Epstein AM (1993) The relation between health insurance coverage and clinical outcomes among women with breast cancer. N Engl J Med 329: 326–331. 8321261 10.1056/NEJM199307293290507

[72] Arndt V, Sturmer T, Stegmaier C, Ziegler H, Dhom G, et al (2001) Socio-demographic factors, health behavior and late-stage diagnosis of breast cancer in Germany: a population-based study. J Clin Epidemiol 54: 719–727. 11438413 10.1016/s0895-4356(00)00351-6

[73] Arndt V, Sturmer T, Stegmaier C, Ziegler H, Dhom G, et al (2002) Patient delay and stage of diagnosis among breast cancer patients in Germany – a population based study. Br J Cancer 86: 1034–1040. 11953844 10.1038/sj.bjc.6600209 PMC2364177

